# Coupling ANFIS with ant colony optimization (ACO) algorithm for 1-, 2-, and 3-days ahead forecasting of daily streamflow, a case study in Poland

**DOI:** 10.1007/s11356-023-26239-3

**Published:** 2023-03-15

**Authors:** Pouya Aghelpour, Renata Graf, Edmund Tomaszewski

**Affiliations:** 1 Department of Water Engineering, Faculty of Agriculture, Bu-Ali Sina University, Hamedan, Iran; 2 Department of Hydrology and Water Management, Institute of Physical Geography and Environmental Planning, Adam Mickiewicz University, Poznań, Poland; 3 Department of Hydrology and Water Management, Institute of Climatology and Hydrology, Faculty of Geographical Sciences, University of Lodz, Łódź, Poland

**Keywords:** Streamflow variation, Neuro-fuzzy, Ant colony optimization, Modeling, Warta river

## Abstract

Finding an efficient and reliable streamflow forecasting model has always been an important challenge for managers and planners of freshwater resources. The current study, based on an adaptive neuro-fuzzy inference system (ANFIS) model, was designed to predict the Warta river (Poland) streamflow for 1 day, 2 days, and 3 days ahead for a data set from the period of 1993–2013. The ANFIS was additionally combined with the ant colony optimization (ACO) algorithm and employed as a meta-heuristic ANFIS-ACO model, which is a novelty in streamflow prediction studies. The investigations showed that on a daily scale, precipitation had a very weak and insignificant effect on the river’s flow variation, so it was not considered as a predictor input. The predictor inputs were selected by the autocorrelation function from among the daily streamflow time lags for all stations. The predictions were evaluated with the actual streamflow data, using such criteria as root mean square error (RMSE), normalized RMSE (NRMSE), and *R*^2^. According to the NRMSE values, which ranged between 0.016–0.006, 0.030–0.013, and 0.038–0.020 for the 1-day, 2-day, and 3-day lead times, respectively, all predictions were classified as excellent in terms of accuracy (prediction quality). The best RMSE value was 1.551 m^3^/s and the highest *R*^2^ value was equal to 0.998, forecast for 1-day lead time. The combination of ANFIS with the ACO algorithm enabled to significantly improve streamflow prediction. The use of this coupling can averagely increase the prediction accuracies of ANFIS by 12.1%, 12.91%, and 13.66%, for 1-day, 2-day, and 3-day lead times, respectively. The current satisfactory results suggest that the employed hybrid approach could be successfully applied for daily streamflow prediction in other catchment areas.

## Introduction

Streamflow forecasting is an important step in aquatic ecosystem management, especially during periods of drought and flooding. The analysis of a series of long-term measurements allows identification of a river’s response to recharge factors and the capture of changes in streamflow over time, which is important for sustainable water use (Firat and Güngör 2007).

There are many methods available for predicting streamflow, and most of them are quite simple to the point that they cannot adequately capture complex relationships between variables (Hussain et al. 2021). Process-based models are preferred in streamflow modeling and forecasting; however, they require a large data set and many calculations (Sharma and Machiwal 2021). Hence, recently, data-driven models (DDM) have become popular. They rely upon the methods of computational intelligence and machine learning (ML) algorithms, and thus assume the presence of a considerable amount of data. In DDM models, a relationship is established between the input and output variables involved in a physical process without explicit knowledge of the physical behavior of the system. The streamflow forecasting process uses multiple (multivariate) linear and nonlinear time series models, which allow for more detailed prediction of streamflow (Fathian 2021).

Due to a high demand for streamflow forecasts, which are used inter alia for the management of water resources in the periods of water excess and water scarcity, attempts are being made to find alternative ways of quick and accurate forecasting of the hydrological regime. The characteristics of the hydrological regime of rivers, including streamflow size and dynamics and water quality, are affected by climatic factors, hydraulic conditions and catchment properties (geological structure, hypsometry, slope of the catchment), as well as human activity mainly related to changes in land use. Due to the complexity of hydrological processes in the catchment area and the complex relationships between the quantitative and qualitative variables of waters, Bayesian network models are also used. Using a Bayesian Network, complex processes can be described in a simple way and provide an approach to incorporate both quantitative and qualitative variables into a single model, as demonstrated by Xu et al. (2019) and Jin et al. (2020). In the Bayesian Networks, each variable only depends on its immediate parent variables, however the networks do not model temporal relationships between variables, meaning they only represent probabilistic relationships between a set of variables at some point in time. Nevertheless, in such areas as streamflow prediction and forecasting using artificial intelligence, the ability to model time relationships is very important.

Increasingly, artificial intelligence methods such as artificial neural networks (ANNs), fuzzy systems (FSs), swarm intelligence (SI), and evolutionary algorithms (EA) have been providing solid tools for estimating hydrological regime parameters. A study by Sharma and Machiwal (2021) presents advances in streamflow forecasting considering both traditional methods and modern research approaches. The ANN techniques, which are currently used on a large scale in streamflow forecasting, have shown good or very good performance in solving this problem (Singh and Deo 2007; Hussain et al. 2021). Concepts, procedures, and applications of artificial neural network models in streamflow forecasting have been presented by Malekian and Chitsaz (2021). Veintimilla-Reyes et al. (2016) have used an artificial neural network to model the runoff and predict the streamflow of the Tume Bomba River in Ecuador, and the obtained results have proven to be very useful in planning flood prevention in the catchment area. The high accuracy of the artificial neural network in streamflow forecasting has been confirmed in studies conducted in arid and semi-arid climates, in Algeria (Achouri et al. 2015), Pakistan (Ghumman et al. 2011) and India (Singh and Deo 2007). The performance of neural network models in streamflow forecasting is often compared to that of linear regression models (Achouri et al. 2015) or stochastic models (Abudu et al. 2010; Valipour et al. 2013; Aghelpour and Varshavian 2020). A comparison of artificial intelligence techniques used in streamflow forecasting has been presented by Firat (2008), while Kasiviswanathan and Sudheer (2013) have conducted a quantification of the predictive uncertainty of artificial neural network-based streamflow forecasting models. Significant prognostic conclusions in this regard can already be established by considering only the daily flow delay as input data. Another group of forecasts takes into account the influence of climatic factors on streamflow changes (Achieng 2021). On a larger scale, hybrid models are used in streamflow forecasting, combining models employing various types of artificial intelligence and machine computation, including metaheuristic algorithms, e.g. the Whale Optimization Algorithm (WOA) or the Grey Wolf Optimizer (GWO) algorithm, which improve the quality of forecasting (Sharma and Machiwal 2021). Hybrid machine learning models for predicting daily runoff from the Naula watershed situated in the upper Ramganga River catchment of Uttarakhand State (India) have been proposed by Malik et al. (2021). Heddam and Kişi (2021) have used a new heuristic extreme learning machine (ELM) model for monthly forecasting of the streamflow in Turkey (the Topluca and Tozkoy stations). The results have confirmed that ELM models can predict monthly streamflow with reliable accuracy comparable to that of multiple linear regression models.

A combination of ANN with the adaptive neuro-fuzzy inference system (FIS) is often used in hydrology to better forecast river regime characteristics (Malekian and Chitsaz 2021). The adaptive network-based fuzzy inference system (ANFIS) approach has been used to construct a streamflow forecasting system. Firat and Güngör (2007) have used ANFIS to forecast streamflow in the Great Menderes River (Turkey), and confirmed that the ANFIS model can be successfully applied and provides high accuracy and reliability in streamflow estimation. In turn, Vafakhah and Janizadeh (2021) have used the ANN and ANFIS systems to estimate event flood peak discharge and runoff volume in the Kasilian catchment (Iran). The results have shown that the ANFIS model performs better than the ANN model in predicting the flood peak streamflow, and also runoff volume. Research conducted in a cool climate, in the Athabasca River in Alberta (Canada), has shown that a sequential ANFIS can accurately predict streamflow with a longer lead time (6 days) by using a single input, compared to nonsequential and multi-input ANFIS (2 days), which ensures accurate predictions of streamflow over large distances and over longer periods of time (Belvederesi et al. 2020). On the other hand, a comparison of the performance of ANFIS and ANN with that of multiple nonlinear regression (MNLR) or stochastic models (e.g., ARIMA) in predicting streamflow in rivers in different climatic zones has shown that the ANFIS model more accurately predicts the daily streamflow (Anusree and Varghese 2016; Dariane and Azimi 2016; Zamani-Sabzi et al. 2007; Ehteram et al. 2019; Poul et al. 2019).

The ANFIS has found application in solving many problems in hydrology, including multivariate drought forecasting (Aghelpour et al. 2021, 2020b), streamflow modeling (Khodakhah et al. 2022; Aghelpour et al. 2022a; Mohammadi et al. 2020), river water temperature prediction (Graf and Aghelpour 2021) or modeling dissolved oxygen concentration (Heddam 2014). Hybrid models of ANFIS with optimization algorithms are often used to automatically adjust ANFIS parameters to reduce learning errors and improve model quality. Bui et al. (2018) have used new hybrids of ANFIS with several optimization algorithms: cultural (ANFIS-CA), bees (ANFIS-BA), and invasive weed optimization (ANFIS-IWO) for flood water susceptibility mapping (FSM) in the Harazhed in Iran. Mundher Yaseen et al. (2017) have developed a novel combination of the ANFIS model with the firefly algorithm as an optimizer tool to construct a hybrid ANFIS-FFA for streamflow forecasting. The results of these studies have shown that not only ANFIS-FFA is superior to a simple ANFIS model, but it also provides a cost-effective modeling framework for predicting streamflow by incorporating fewer input variables required for relatively better performance. The use of optimization algorithms not only improves the performance of models used in streamflow forecasting, but also of those used to predict extreme phenomena, such as flood or drought. Aghelpour et al. (2020a) have applied ANFIS combined with bio-inspired optimization algorithms: ANFIS-ACO (ANFIS combined with ant colony optimization), ANFIS-GA (ANFIS combined with GA) and ANFIS-PSO (ANFIS combined with PSO) for drought forecasting. It has been shown that the best results in terms of ANFIS optimization are obtained for the ACO and GA algorithms, which have improved the performance of the model by approximately 46% and 43%, respectively.

This study proposes a novel methodology to tackle problems related to streamflow forecasting. The paper investigates the possibility of using ANFIS as a simple machine learning model, and its hybridized type with the ACO bio-inspired algorithm as a meta-heuristic model for forecasting daily streamflow. The streamflow forecast was presented for three-time horizons: of 1 day, 2 days, and 3 days in advance. The assessment of the application and application possibilities of ANFIS and ANFIS-ACO was presented with regard to the Warta river catchment, the third longest river in Poland, constituting an economically and environmentally important river system. A significant problem observed in the Warta Water Region is its exposure to atmospheric and hydrological drought and the associated high streamflow sensitivity, which manifests itself in its significant reduction, especially in periods of prolonged summer and autumn drought. An additional problem is the accumulation of pressure related to the excessive surface water abstraction for crop irrigation purposes in the periods of low streamflow, which adversely affects the minimum acceptable flow, i.e. the minimum amount of water flow necessary to sustain life in the river. For the above reasons, it is extremely important to develop an accurate predictor model for this catchment area. The current study is conducted for the first time on the Warta river catchment. The ANFIS model and its hybridized version with the ACO algorithm were used to estimate and predict hydrometeorological components (such as solar radiation, droughts, evapotranspiration etc.). It was the first time that they were applied and evaluated in the prediction of daily river streamflow, especially in a multi-step ahead prediction study.

## Materials and methods

### Study catchment

**Fig. 1:**
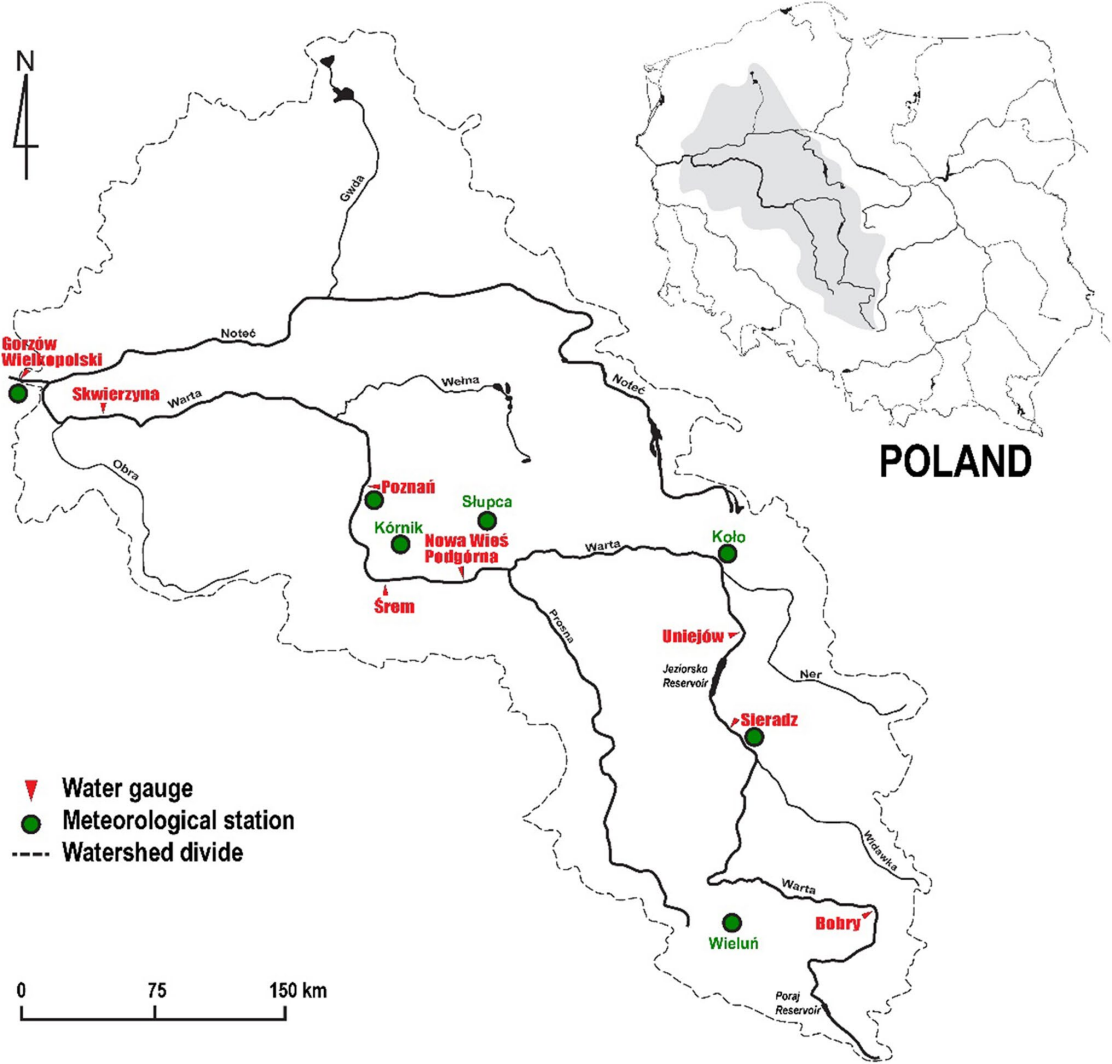
Study area and the location of water gauges and meteorological stations of the Institute of Meteorology and Water Management–National Research Institute (IMGW-PIB, Warsaw, Poland)

The Warta river catchment area is located in the western part of Poland, situated in Central Europe (Fig. 1). This river is the right tributary of the Odra River and the third longest (808 km) river in Poland. In terms of water management, it forms the so-called Warta Water Region (with an area of 54,500 km^2^), which covers approximately 17.4% of the country’s area.

In the land use structure in the region, arable land prevails, accounting for approximately 60% of the area, mainly located in its central part. Forests account for approximately 30% in the Warta River catchment area. The largest forests are found mainly in the north-western part of the area, where the forest cover rate often reaches 50%. Grassland (meadows and pastures) occupies a much smaller area compared to arable land and forests. It mainly occurs in the valleys of the middle Warta River and its larger tributaries, the Noteć and the Obra rivers (Fig. 1). Urbanized areas account for only 8% of the catchment area.

The Warta river catchment area belongs to nine climatic regions, as determined by Woś (2010). The average annual air temperature ranges from 7.5 °C in the north to 8.5 °C in the west of the study area. In January, the coldest month, the average temperature ranges from − 1.2 °C (in the west) to − 2.5 °C (in the south-east), while in July, the warmest month of the year, the average temperature ranges from 16.9 °C (in the north) to 18.1 °C (west and south). Annual rainfall totals in the study area range between 520 mm in the Kujawy region (in the north-east) and 675 mm in the south of the catchment area. The highest monthly sums of precipitation are recorded in July and range from 71 mm (in the west) to 96 mm in the south of the catchment area. The lowest monthly rainfall totals occur in February, ranging from 26 mm (southwest and central part of the region) to 33 mm in the south. About 60% of the area receives less than 550 mm of rainfall annually (compared to the annual average for Poland of 600 mm). Precipitation less than 500 mm occurs in the central strip of the region from the Poznań Lake District to Kujawy.

In the analyzed section of the Warta river, regional differentiation of the hydrological regime characteristics is observed—from a medium (upper and bottom reaches) to a highly (from Nowa Wieś Podgórna to Poznań) developed nival regime (Wrzesiński and Perz 2016). The Warta river catchment area is characterized by a large diversity of the total runoff height in individual catchments. The runoff of the rivers in the southern and northern parts of the catchment is 200–300 mm, while the runoff of the rivers in the central part is below 150 mm, and locally even below 80 mm. The Warta river is dominated by early spring high streamflow periods, caused by the release of water from melting snow cover and frozen soil. Periods of high-streamflow in the summer (caused by rainfalls) are of secondary importance, as they are irregular, even though the flow may be higher than the spring one. The Warta River catchment area is characterized by the largest area exposed to flooding risk in the Odra River basin, while the flood phenomena may have a different origin (snowmelt and ice-jam floods, intense and long-lasting rainfall). The Warta Water Region has the greatest area and time diversity of low streamflow. In the period between July–August and September–October, summer low streamflow is observed, which usually lasts long and often constitutes an essential indicator of the development of hydrological drought. Small runoff deficits may occur as early as May, while the maximum runoff deficits occur in August (Kozek and Tomaszewski 2018). Low streamflow in winter is rare and it is usually an extension of the summer—autumn low streamflow.

There are two storage reservoirs along the Warta River: Poraj (upper reaches of the river) and Jeziorsko (middle reaches of the river) (Fig. 1). The main purpose of the Poraj reservoir is to ensure stable water supply to the “Huta Częstochowa” steelworks by equalizing the flow of the Warta River. Besides, it has a recreational function, offering the opportunity to practice water sports. Below the town of Sieradz, there is another large water reservoir, Jeziorsko, with an area of 42.3 km^2^, with a dam in Skęczniewo, built in 1986. The river was fully dammed in 1992 (Gorączko and Pawłowski 2014). The Jeziorsko reservoir is mainly used for energy and agricultural purposes. The Poraj and Jeziorsko reservoirs located on the Warta river provide flood protection in the river catchment area.

The Warta river is of great economic importance and performs important natural and landscape functions. First of all, it provides the inhabitants of the Wielkopolska region with water supply security. The economic potential of the river is mainly related to water abstraction for industrial and agricultural purposes, which is guaranteed by maintaining an appropriate level of available resources based on the streamflow (RZGW 2007).

### Dataset

In order to forecast streamflow, daily data from the period from 01.11.1993 (01.01.1994 in the hydrological year) to 31.10.2013 (31.12.2013 in the hydrological year) were used for 8 water gauges located along the Warta river (Fig. 1). Additionally, daily data on precipitation in the studied region from 4 meteorological (precipitation) stations located in the catchment area were included in the forecasting process. Data were presented in relation to the hydrological year, which in Poland lasts from 1 November until 31 October. Coordinates of the stations and the dataset’s details are given in Table 1.

**Table 1:** The meteorological and hydrometric stations’ coordinates and the data details during the study period, 1994–2013 hydrological years (equal to 01/11/1993–31/10/2013). The rows of hydrometric stations show the details of daily streamflow dataset in m^3^/s

Station						Dataset*				
Type	Name	Catchment area [km^2^]	Coordinates			Max	Mean	Min	STD	Skew
			Latitude	Longitude	Altitude					
Hydrometric station (daily streamflow dataset [${m}^{3}/s$])	Bobry	1800.5	51^o^01′30"	19^o^24′30"	205.50	10.89	7.21	2.60	137.00	4.08
	Sieradz	8139.6	51^o^36′00"	18^o^44′40"	125.10	46.94	28.50	16.60	408.00	3.52
	Uniejów	9203.1	51^o^58′40"	18^o^47′00"	101.98	50.72	26.86	18.00	362.00	3.76
	Nowa Wieś	20,762.8	52^o^08′30"	17^o^35′30"	67.04	96.03	54.66	30.70	630.00	2.40
	Śrem	22,434.2	52^o^05′35"	17^o^01′05"	57.84	100.69	58.00	33.20	597.00	2.08
	Poznań	25,910.9	52^o^23′55"	16^o^56′35"	49.46	108.47	61.52	37.50	630.00	2.02
	Skwierzyna	32,053.7	52^o^36′10"	15^o^30′10"	21.82	130.69	74.42	42.20	592.00	1.81
	Gorzów Wielkopolski	52,404.3	52^o^43′45"	15^o^14′30"	15.51	211.84	103.10	65.40	899.00	1.59
Meteorological station (daily precipitation dataset [*mm*])	Wieluń	‘x’	51^o^12′40"	18^o^33′28"	185.0	4.25	1.75	0.00	78.70	5.55
	Koło	‘x’	52^o^12′01"	18^o^39′41"	109.0	3.81	1.56	0.00	56.50	4.87
	Poznań	‘x’	52^o^25′02"	16^o^50′09"	91.0	3.92	1.58	0.00	85.70	6.23
	Gorzów Wielkopolski	‘x’	52^o^44′28"	15^o^16′38"	64.0	3.77	1.63	0.00	70.80	5.01

^*^ *Max.* maximum, *Min.* minimum, *STD*. standard deviation, *Skew.* skewness

The aforementioned data are held at the Institute of Meteorology and Water Management—National Research Institute (IMWM-NRI, Warsaw, Poland) and are available at https://danepubliczne.imgw.pl*.*

### Adaptive neuro-fuzzy inference system (ANFIS)

Owing to the fact that ANFIS is a combination of ANN and FIS, the FIS parameters are identified by ANN learning algorithm (Jang et al. 1997; Nourani et al. 2016). In ANFIS, the FIS parameters are calculated using the back-propagation algorithm. This system is FIS dependent, reflects ambiguous information, and has fuzzy IF–THEN rules (Kisi et al. 2017). According to Jang et al. (1997), ANFIS is able to estimate real continuous functions on a compact set of parameters. The ANFIS learning principle that defines its parameters is a combination of backpropagation and least squares methods (Fallah-Mehdipour et al. 2014).

**Fig. 2:**
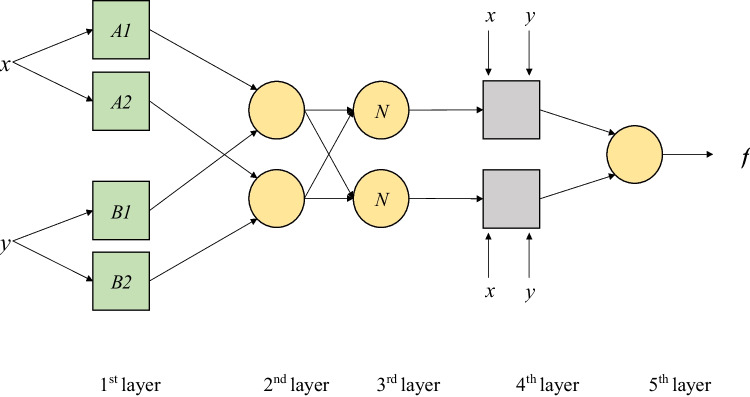
Schematic structure of the ANFIS model with two inputs (according Aghelpour et al. 2022a, b)

Intelligent control such as fuzzy logic is a suitable choice for controlling nonlinear systems. The input values of the model can include numerical measurement data of various physical quantities. The main task in constructing a fuzzy model is to determine the rule base and the number of fuzzy sets (membership functions) assigned to the individual inputs and outputs of the model. Subsequently, it is also necessary to properly select the algorithms for aggregating simple premises. When constructing fuzzy models, a rule base (knowledge base) is often obtained by automatically extracting rules based on the input and output numerical data of the modeled phenomenon or process. Considering the ANFIS structure, the overall output is a linear combination of the consequent parameters. The final output *f*_out_ can be written as (Çaydas et al. 2009):

1 $$c{f}_{out}={\overline{\omega }}_{1}{f}_{1}+{\overline{\omega }}_{2}{f}_{2}=\frac{{\omega }_{1}}{{\omega }_{1}+{\omega }_{2}}{f}_{1}+\frac{{\omega }_{2}}{{\omega }_{1}+{\omega }_{2}}{f}_{2}=\left({\overline{\omega }}_{1}x\right){p}_{1}+\left({\overline{\omega }}_{1}y\right){q}_{1}+\left({\overline{\omega }}_{1}\right){r}_{1}+\left({\overline{\omega }}_{2}x\right){p}_{2}+\left({\overline{\omega }}_{2}y\right){q}_{2}+\left({\overline{\omega }}_{2}\right){r}_{2}$$

where: ${\omega }_{i}$ (output) represents the firing strength of a rule, ${f}_{1}$ and ${f}_{2}$ are the fuzzy rules, $x$ and $y$ are the input nodes of ANFIS, and ${p}_{i}$, ${q}_{i}$ and ${r}_{i}$ are the parameters set (referred to as the consequent parameters). Figure 2 shows a schematic diagram of ANFIS modeling processes.

The most common method of automatic extraction of rules from numerical data is grouping of input/output data, often called clustering. The effective operation of clustering algorithms enables the determination of the centers of gravity of the clusters, i.e. the areas of concentration of the measurement data. It is connected with determining the parameters of the fuzzy model together with the organization of its structure (rule base). This method can be used, for example, to build a preliminary version of a fuzzy model, when there is no rule base, but only numerical data reflecting the modeled process, phenomenon or object.

The FIS usually uses two methods of inference: the Mamdani method (Mamdani and Assilian 1975) and the Sugeno method (Takagi and Sugeno 1985), and the latter was employed in the present study. Inference in the neuro-fuzzy system takes place in several stages, which are carried out by an appropriate layer of the neural structure or the like. The first layer recreates the membership functions of fuzzy sets contained in the assumptions of the rules. The second layer performs the operations of intersecting fuzzy sets using the algebraic product. The third and fourth layers perform operations related to the sharpening of the resulting membership function.

#### Fuzzy C-Means (FCM) clustering method

Data grouping or data clustering is a data mining concept. Clustering is a method of unsupervised learning, the purpose of which is to distinguish groups of data, i.e., to divide a set of elements into subsets (clusters, data clusters) in order that the elements assigned to individual clusters be similar to each other, and the clusters differ from each other as much as possible. Data can form clusters of various sizes, shapes, or densities. They can be well separated from each other, be connected to each other or overlap. A cluster is usually represented by its central point (center). The similarity of data set elements assigned to a selected cluster is described by the measure of the distance between the elements and the central point (center) of the cluster.

Fuzzy C-Means clustering is a soft clustering method in which each data point belongs to a cluster with a membership degree (Dunn 1973; Bezdek 1981). The fuzzy clustering method consists in allocating elements to more than one cluster with membership probability. One dataset element can belong to several clusters, to each of them to some extent. The fuzzy C-Means (FCM) is a fuzzy form of the C-means algorithm that does not consider the sharp boundaries between the clusters (Velmurugan 2014; Jain 2010). In this way, the remarkable advantage of the FCM is in helping to assign partial items from each object to different groups in a global set rather than being wholly owned by a group (Abdulshahed et al. 2015). Fuzzy grouping is helpful when there is no clear boundary between groups of objects.

FCM clustering is viewed as an optimization problem that tries to optimize the objective function (Fattahi et al. 2016):

2 $${J}_{FCM}= \sum\limits_{i=1}^{C}\sum\limits_{j=1}^{n}{u}_{ij}^{m}{d}_{y}^{2}$$

where: *C* is the number of clusters, *u*_*ij*_ ∈ [0,1] expresses the membership degree of the data point *xj* belonging to the *i*^*th*^ fuzzy group, *d*_*ij*=_ *w*_*i*_* – x*_*j*_ is the Euclidean distance between the *i*^*th*^ cluster center *w*_*i*_ and *j*^*th*^ data point *x*_*j*_, and *m* ∈ (1, ∞) is a weighting exponent that influences the fuzziness of the clusters.

By iteratively updating the cluster centers and the membership grades for each data point, the FCM iteratively moves the cluster centers to the right location within a data set. This iteration is based on minimizing an objective function that represents the distance from any given data point to a cluster center weighted by the membership grade of that data point.

The ANFIS model contains two sets of parameters, i.e. premise and consequence parameters. Premise parameters are input parameters of the membership functions and their purpose is to specify the shape and location of the input membership functions (parameters of input MFs), and consequence parameters are the parameters of output membership functions (Jang 1993). To estimate these parameters, simple ANFIS uses the least square (or gradient decent) algorithm; but in a hybridized form of ANFIS, the complex optimization algorithm is used for this purpose (Aghelpour et al. 2022b; Kisi et al. 2019). In the current study, the ACO does this task for ANFIS in a hybridized meta-heuristic form. This algorithm is described in the next section.

### Ant colony optimization (ACO) algorithm

Optimization problems usually relate to the search for and finding of the best solutions (paths) in various types of models and calculations, or solutions to problems of scheduling, routing or group work. The ACO algorithm belongs to the group of metaheuristic algorithms. It is a probabilistic technique for solving problems by looking for good paths in graphs and finding optimal paths. It was inspired by the behavior of ants searching for food for their colony (Dorigo et al. 1996). In the ACO algorithm, optimization is carried out iteratively by a number of agents who communicate using messages left in the environment and randomly search the space, favoring solutions indicated by such messages. At each iteration, ACO generates global ants and calculates their fitness. The continuous ACO is based on both local and global search.

The ACO algorithm was inspired by the foraging behavior of an ant colony (indirect foraging communication, represented by pheromone trace) and developed for discrete optimization problems. It has been described as an algorithm that can find the best route through a graph. For a dynamically changing graph, the ACO algorithm can run continuously and adapt to changes in real time. It is successfully used to solve discrete linear tasks, it solves the traveling salesman problem, the problem of square allocation (QAP), task scheduling, load balancing of communication links, as well as grouping, searching, sequential sorting or packet routing in networks.

The two most important formulas that cover the ant systems problem are: “path selection” and “pheromone update” (Modrzejewski 2009). According to the “path selection” formula, the ant will travel from point i to point j with a probability of:

3 $${p}_{i, j}= \frac{{\tau }_{i, j }^{\alpha }{\eta }_{i, j}^{\beta }}{\sum {\tau }_{i, j}^{\alpha } {\eta }_{i, j}^{\beta }}$$

where: ${\tau }_{i, j}$ is the amount of pheromone in path *i, j*; *α* is the parameter to control the influence of ${\tau }_{i, j}$*; η*_*i, j*_ determines the attractiveness of the path *i, j* and *β* is the parameter to control the influence of *η*_*i, j*_*.*

**Fig. 3:**
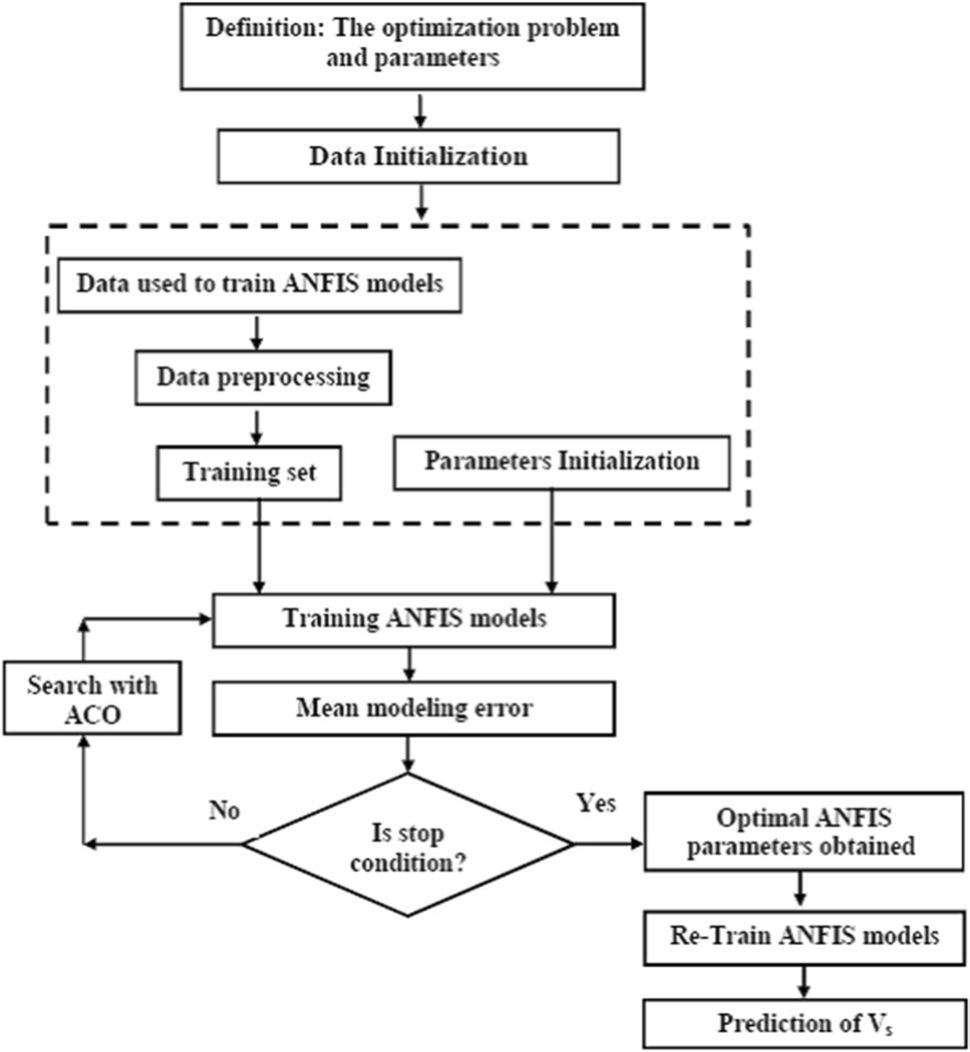
The process of optimizing ANFIS parameters with the ACO (according Fattahi et al. 2016)

While *"pheromone update"* is represented by the formula:

4 $${\tau }_{i, j}^{\left(n\right)}= {p\tau }_{i, j}^{(s)}+\Delta {\tau }_{i, j}$$

where: ${\tau }_{i, j}$ is the amount of pheromone in path *i, j*; *p* is the pheromone evaporation scale and $\Delta {\tau }_{i, j}$ is the amount of pheromone left. The choice of the ACO algorithm entails ensuring the automatic adjustment of ANFIS parameters to reduce learning errors and improve the model’s performance. The process of optimizing ANFIS parameters with the ACO is shown in Fig. 3.

The ACO algorithm has four operators, namely population, maximum number of iterations, intensification factor, and deviation-distance ratio (as presented in Table 2). These factors have been examined and determined by a trial–error method (Khosravi et al. 2018; Halabi et al. 2018; Kisi et al. 2019; Aghelpour et al. 2020a). The finally selected ACO operators are shown in Table 2.

**Table 2:** Selected operators for ant colony optimization

Parameter	Value
Population	100
Maximum number of iterations	200
Intensification factor (selection pressure)	0.5
Deviation-Distance ratio	1

### Evaluation measurements

**Fig. 4:**
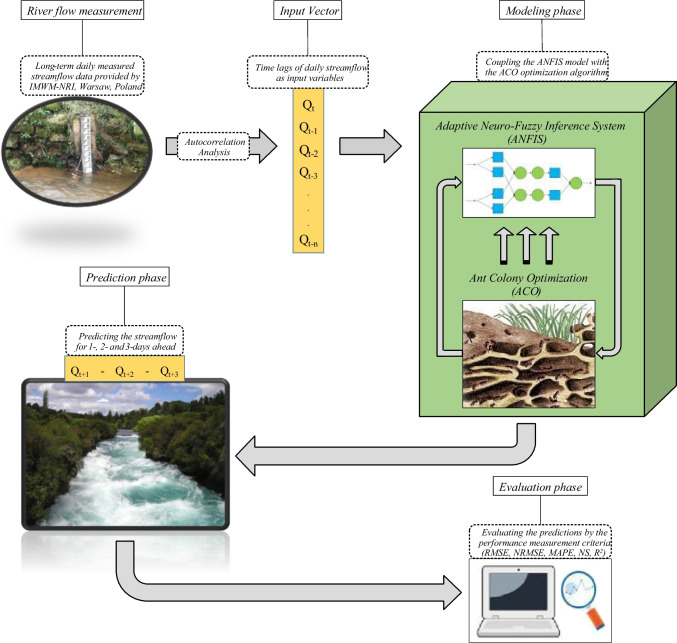
General flowchart of the current study’s modeling and prediction process

In the present study, evaluation metrics were used to evaluate the accuracy of daily streamflow prediction models. To calculate these criteria, two series of observed and predicted datasets are required. The criteria used in the present study include root mean square error (RMSE), normalized RMSE (NRMSE), mean absolute percentage error (MAPE), Nash-Sutcliff efficiency (NSE), and coefficient of determination (*R*^2^). Their equations are listed below:

5 $$RMSE=\sqrt{\frac{1}{n}\sum\limits_{i=1}^{n}{\left({QO}_{i}-{QP}_{i}\right)}^{2}}$$

6 $$NRMSE=\frac{\sqrt{\frac{1}{n}\sum\nolimits_{i=1}^{n}{\left({QO}_{i}-{QP}_{i}\right)}^{2}}}{{QO}_{max}-{QO}_{min}}$$

7 $$MAPE=\frac{1}{n}\sum_{i=1}^{n}\frac{\left|{QO}_{i}-{QP}_{i}\right|}{{QO}_{i}}\times 100$$

8 $$NSE=1-\frac{\sum\nolimits_{i=1}^{n}{\left({QO}_{i}-{QP}_{i}\right)}^{2}}{\sum\nolimits_{i=1}^{n}{\left({QO}_{i}-\overline{QO }\right)}^{2}}$$

9 $${R}^{2}={\left[\frac{{\sum\nolimits _{i=1}^n}\left({QO}_{i}-\overline{QO }\right)({QP}_{i}-\overline{QP })}{\sqrt{{\sum\nolimits _{i=1}^n}{({QO}_{i}-\overline{QO })}^{2}}\times \sqrt{{\sum }_{i=1}^{n}{({QP}_{i}-\overline{QP })}^{2}}}\right]}^{2}$$

where: ${QO}_{i}$ and ${QP}_{i}$ are the observed and predicted daily streamflow values on the ${i}^{th}$ day, respectively;$\overline{QO }$ and $\overline{QP }$ denotes the average values of observed and predicted streamflow, respectively; ${QO}_{max}$ and ${QO}_{min}$ are the maximum and minimum values of observed streamflow, respectively; and $n$ refers to the number of modeling samples (here, the total number of days under study). The closer the RMSE, NRMSE, and MAPE values ​​are to zero, and the closer the NSE and *R*^2^ values are to one, the better the model performance. NRMSE values are also classified into 4 groups: NRMSE > 0.3; 0.2 < NRMSE < 0.3; 0.1 < NRMSE < 0.2; and NRMSE < 0.1; which indicate poor, moderate, good and excellent performance of the models, respectively (Bahrami-Pichaghchi and Aghelpour 2022; Aghelpour and Norooz-Valashedi 2022). The general step of the current study is illustrated as a flowchart in Fig. 4.

## Results

In this section, first the models’ inputs will be selected. Then, the ANFIS and ANFIS-ACO models will be implemented and evaluated for 1-step, 2-steps, and 3-steps ahead forecasting. The models will be then compared to each other, and finally, the prediction accuracy will be evaluated along the river at the hydrometric stations.

### Input selection

**Fig. 5:**
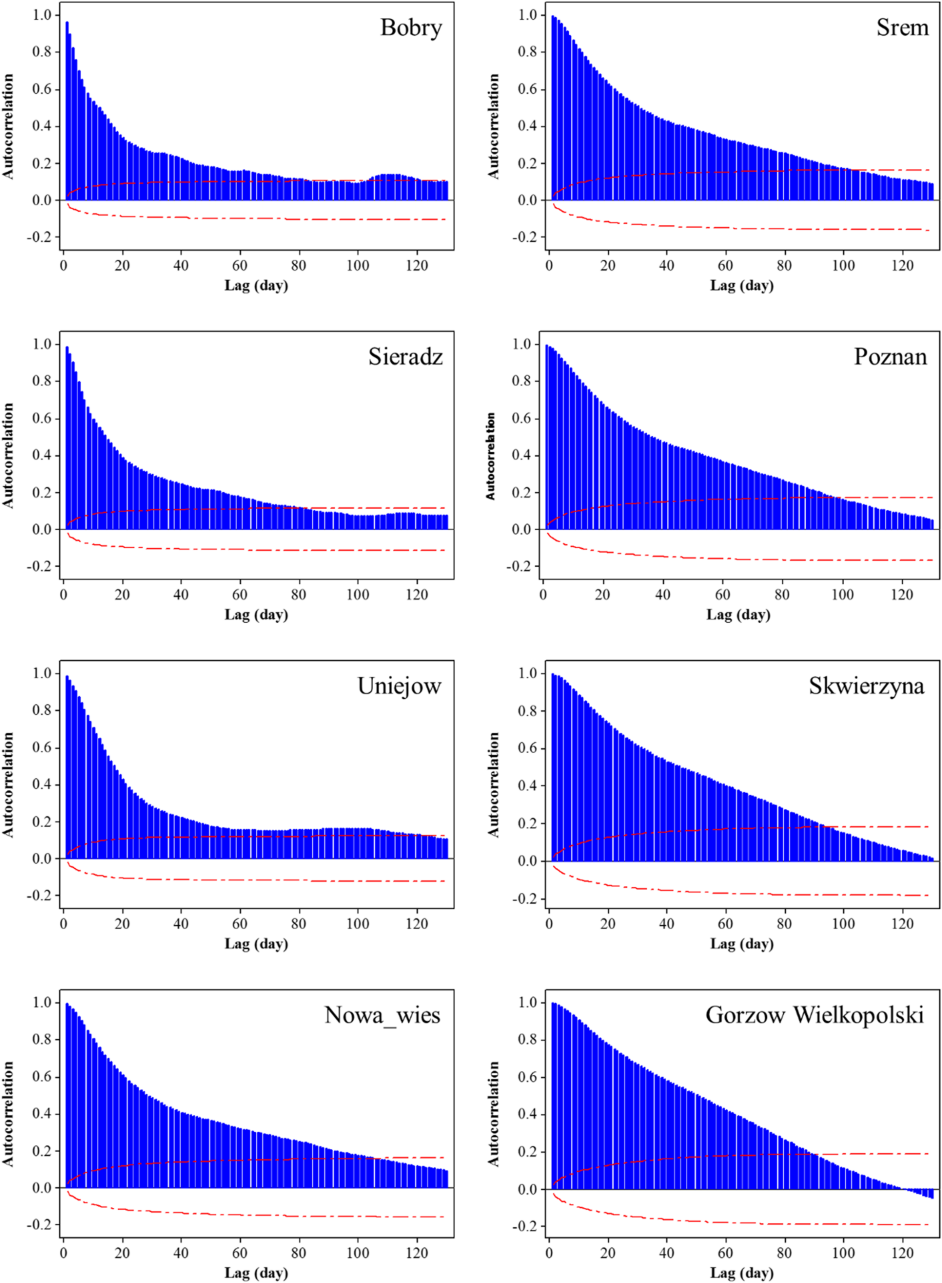
Autocorrelation function graphs for the streamflow in hydrometeric stations

**Fig. 6:**
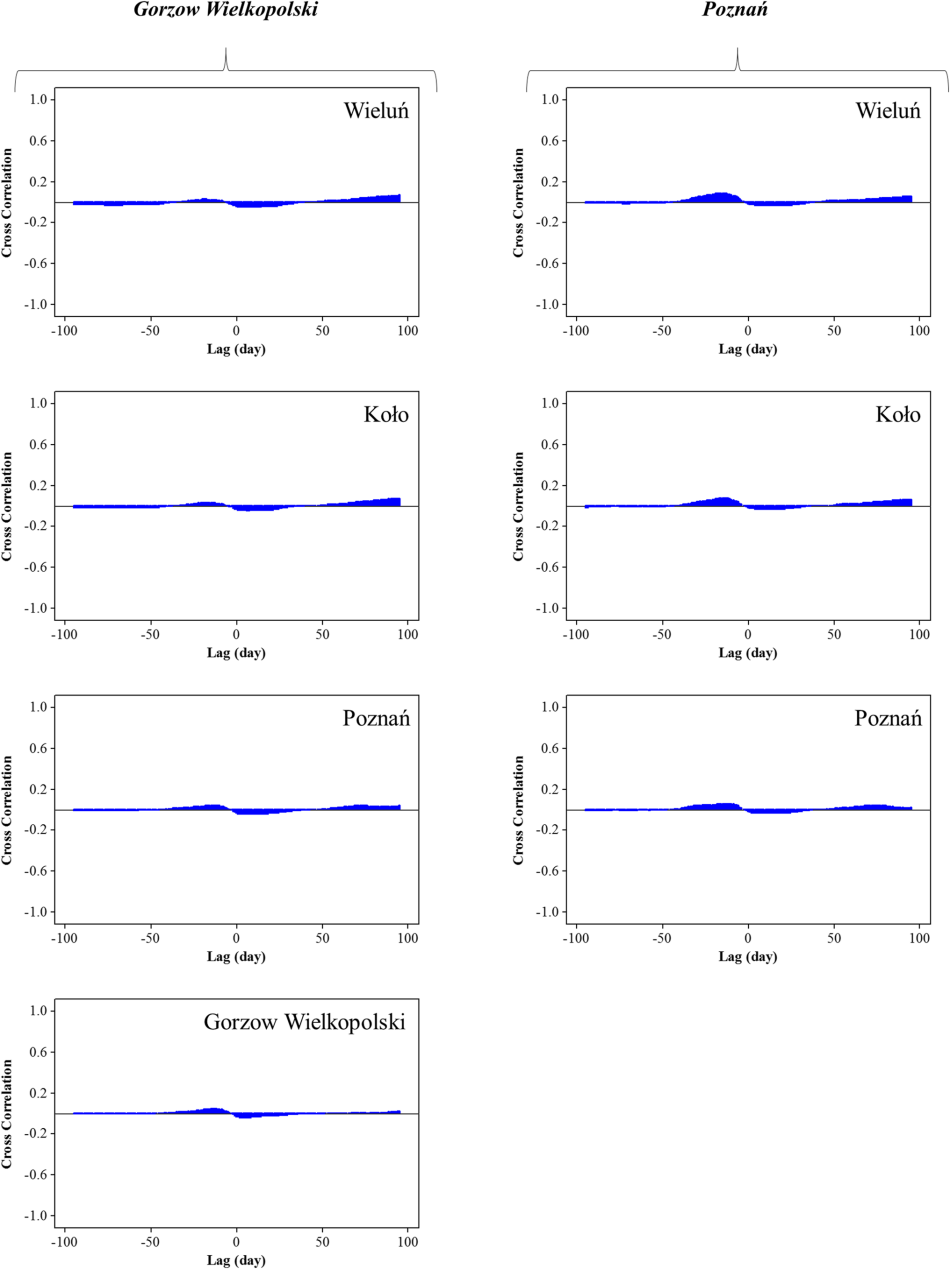
Cross-correlation graphs between the daily recorded streamflow data of Gorzów Wielkopolski and Poznań hydrometric stations and their upstream recorded precipitation data of the meteorological stations (Wieluń, Koło, Poznań and Gorzów Wielkopolski)

The models’ input variables were selected from among the daily time lags of streamflow and precipitation. The Autocorrelation Function (ACF) plots were used to analyze the streamflow time lags (Fig. 5), and the cross-correlation graphs (Fig. 6) were analyzed to investigate the effectiveness of precipitation time lags.

As is obvious from Fig. 5, there is strong autocorrelation between the time lags of streamflow in all stations. The correlations are high in the small time lags and they decrease with increasing time lags. The significance continues up to 80 or in some cases even 100 daily time lags; but the strongest correlations exist in the primary time lags, so it is reasonable to use these time lags among the streamflow time lags. In Fig. 6, correlations of daily precipitation time lags are observable, and the negative time lags show the impact of precipitation on streamflow.

Figure 6 examines the effectiveness of precipitation in the meteorological stations located in the upper basin on the streamflow of the Poznań and Gorzów Wielkopolski hydrometric stations. The graphs show that the precipitation values at all upper points (including Wieluń, Koło and Poznań for Poznań station’s streamflow, and Wieluń, Koło, Poznań and Gorzów Wielkopolski for Gorzów Wielkopolski station’s streamflow) do not show strong cross-correlations with the streamflow. The cross-correlations are under 0.2 and they are weaker compared to the streamflow time lags (in most cases, bigger than 0.9). Therefore, using the precipitation lags as inputs increases the prediction error for the models. The reason for this weak correlation can be associated with the time scale of this study. The impact of precipitation on streamflow can be observed after some hours or even minutes; hence, the precipitation time lags can be used for hourly or momentary streamflow forecasting, while the purpose of the current investigation was to predict daily streamflow. Eventually, the primary time lags of streamflow were used for the prediction. In accordance with parsimony, it was decided to use only three time lags for each forecasting horizon. For example, in the Bobry station (Fig. 5), the first three time lags (1, 2 and 3) had correlations higher than 0.9, while the autocorrelation of the 4^th^ time lag was around 0.75. This means that the use of the 4^th^ time lag can significantly reduce prediction accuracy. Moreover, according to the principle of parsimony, in situations where changes in the model accuracy are minor, the smaller the number of input variables, the more reasonable it is. Consequently, 1-, 2-, and 3-day time lags were used for 1-day ahead forecasting, 2-, 3-, and 4-day time lags for 2-days ahead forecasting and 3-, 4-, and 5-day time lags for 3-days ahead streamflow forecasting.

### Modeling and evaluation of the predictions

The ANFIS and ANFIS-ACO models were implemented based on the defined input matrixes. The regularization parameters of ANFIS are premise and consequence parameters. The simple ANFIS model used the least square method for optimizing these two sets of parameters while the hybridized ANFIS-ACO model utilized the ant colony optimization algorithm for this purpose. The evaluation results are shown in Tables 3, 4, and 5, for the 1-, 2-, and 3-days ahead forecasting of streamflow. It is worth mentioning that the discussions are around the testing phase, which shows the prediction validity of the models.

**Table 3:** Evaluating the 1-day ahead predictions

Station	ANFIS						ANFIS-ACO					
	Train			Test			Train			Test		
	RMSE	MAPE	NS	RMSE	MAPE	NS	RMSE	MAPE	NS	RMSE	MAPE	NS
	(${m}^{3}/s$)	(%)		(${m}^{3}/s$)	(%)		(${m}^{3}/s$)	(%)		(${m}^{3}/s$)	(%)	
Bobry	1.434	6.591	0.957	2.076	5.852	0.933	1.551	7.332	0.950	1.882	6.156	0.945
Sieradz	2.742	2.474	0.990	3.517	2.248	0.987	2.777	2.817	0.990	3.082	2.721	0.990
Uniejów	3.797	3.562	0.975	3.941	3.101	0.986	4.029	3.564	0.972	3.402	2.881	0.990
Nowa Wieś	2.835	2.278	0.997	4.289	2.006	0.996	3.008	2.415	0.996	3.947	1.977	0.997
Śrem	2.696	1.989	0.997	4.345	1.595	0.996	2.925	2.113	0.997	3.568	1.571	0.997
Poznań	2.377	1.731	0.998	3.601	1.599	0.998	2.518	1.827	0.998	3.350	1.534	0.998
Skwierzyna	2.415	1.390	0.999	5.081	1.576	0.997	2.508	1.438	0.999	4.610	1.551	0.997
Gorzów Wielkopolski	3.199	1.227	0.999	5.355	1.208	0.998	3.332	1.272	0.999	4.937	1.187	0.998

**Table 4:** Evaluating the 2-days ahead predictions

Station	ANFIS						ANFIS-ACO					
	Train			Test			Train			Test		
	RMSE	MAPE	NS	RMSE	MAPE	NS	RMSE	MAPE	NS	RMSE	MAPE	NS
	(${m}^{3}/s$)	(%)		(${m}^{3}/s$)	(%)		(${m}^{3}/s$)	(%)		(${m}^{3}/s$)	(%)	
Bobry	2.321	10.369	0.888	3.956	9.944	0.756	2.671	11.972	0.851	3.534	10.758	0.806
Sieradz	5.548	4.849	0.960	7.148	5.270	0.945	5.872	5.628	0.955	6.366	5.525	0.956
Uniejów	5.970	6.099	0.939	6.941	5.381	0.957	6.266	6.142	0.933	6.312	5.453	0.965
Nowa Wieś	5.465	4.159	0.987	8.819	3.765	0.984	5.809	4.463	0.986	8.217	3.680	0.986
Śrem	5.256	3.800	0.990	8.926	3.240	0.983	5.733	4.107	0.989	7.951	3.183	0.987
Poznań	4.416	3.162	0.994	8.630	3.155	0.988	4.783	3.418	0.992	7.320	2.998	0.991
Skwierzyna	4.488	2.573	0.996	9.747	3.082	0.988	4.732	2.700	0.995	8.323	2.992	0.992
Gorzów Wielkopolski	5.927	2.257	0.996	11.558	2.355	0.990	6.294	2.384	0.996	10.094	2.286	0.992

**Table 5:** Evaluating the 3-days ahead predictions

Station	ANFIS						ANFIS-ACO					
	Train			Test			Train			Test		
	RMSE	MAPE	NS	RMSE	MAPE	NS	RMSE	MAPE	NS	RMSE	MAPE	NS
	(${m}^{3}/s$)	(%)		(${m}^{3}/s$)	(%)		(${m}^{3}/s$)	(%)		(${m}^{3}/s$)	(%)	
Bobry	3.093	13.606	0.800	5.015	13.678	0.608	3.572	15.963	0.734	4.732	14.725	0.651
Sieradz	8.368	7.423	0.909	10.296	7.105	0.886	8.741	7.499	0.901	9.512	6.930	0.902
Uniejów	7.779	8.295	0.896	11.013	7.950	0.893	8.125	8.368	0.887	9.231	7.815	0.925
Nowa Wieś	8.238	5.995	0.971	13.443	5.613	0.962	8.699	6.461	0.968	12.647	5.481	0.967
Śrem	7.986	5.609	0.978	14.643	5.014	0.955	8.714	6.085	0.974	12.249	5.010	0.969
Poznań	6.648	4.674	0.985	14.433	4.819	0.965	7.248	5.087	0.983	11.894	4.634	0.976
Skwierzyna	6.747	3.799	0.990	14.594	4.609	0.974	7.156	4.030	0.989	12.323	4.475	0.981
Gorzów Wielkopolski	8.872	3.298	0.992	17.360	3.518	0.977	9.437	3.506	0.991	15.757	3.479	0.981

Table 3 illustrates the 1-day ahead forecasting evaluation. According to the values of the RMSE, MAPE, and NS evaluation criteria, both models are well evaluated in all stations. Among the stations, the lowest RMSE belongs to the ANFIS-ACO model at the Bobry station (1.882 m^3^/s) and the highest RMSE belongs to the simple ANFIS model in the Gorzów Wielkopolski station (5.355 m^3^/s). Both the lowest and the highest MAPE values belong to the ANFIS-ACO model in the Gorzów Wielkopolski and Bobry stations (1.187% and 6.156%), respectively. The NS values are higher than 0.90 which shows that the 1 step ahead forecasting can be excellently represented by both models.

Evaluation of the 2 days ahead forecasting of the streamflow is shown in Table 4. The NS values are bigger than 0.75, which indicates that the models have a good performance in 2 days ahead forecasting. The highest and lowest values of the RMSE criterion are 3.534 m^3^/s in the Bobry station and 11.558 m^3^/s in the Gorzów Wielkopolski station, and they belong to the ANFIS-ACO and ANFIS models, respectively. The lowest and the highest MAPE values belong to ANFIS-ACO, and they are equal to 10.758% in Gorzow Wielkopolski and 2.286% in the Bobry station.

Table 5 shows the prediction evaluation metrics concerning the 3 days ahead forecasting horizon. Based on the values of the RMSE, MAPE and NS evaluation criteria, the models are moderately evaluated in the stations. The NS values are higher than 0.6, which indicates a moderate performance of the prediction model. The highest NS is recorded for ANFIS-ACO in the Gorzów Wielkopolski station (NS = 0.981) and the lowest for the simple ANFIS model in the Bobry station (NS = 0.608). The best RMSE values belong to the Bobry station and are equal to 5.015 and 4.732 m^3^/s for the ANFIS and ANFIS-ACO models, respectively. The best MAPE values are also recorded at the Gorzów Wielkopolski station (5.015 m^3^/s for ANFIS and 4.732 m^3^/s for ANFIS-ACO).

### Comparing the models

**Fig. 7:**
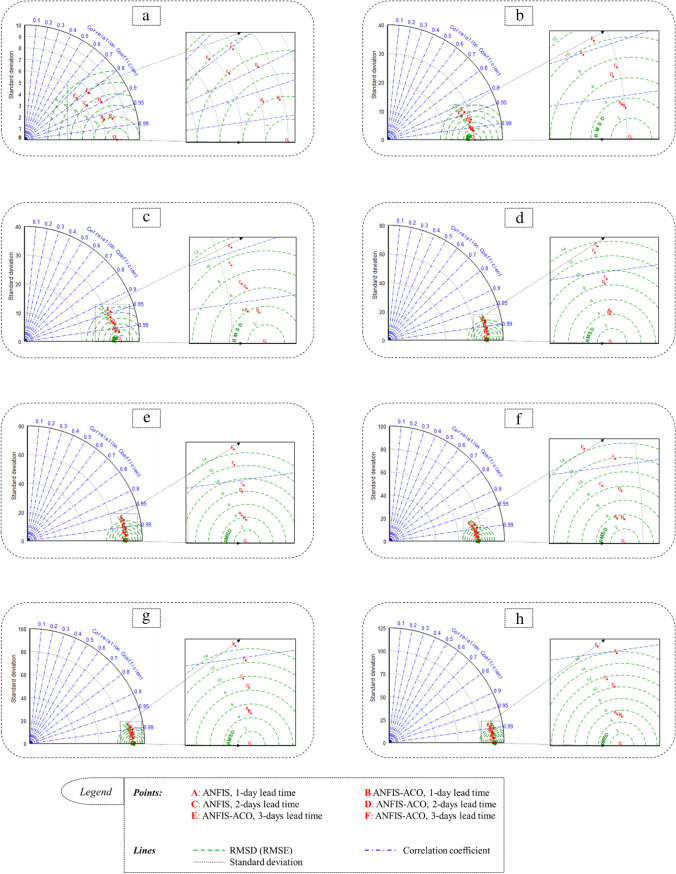
Taylor diagrams to evaluate the models’ prediction accuracy in each station. The small letters on the right side refer to the hydrometric stations, including Bobry (**a**), Sieradz (**b**), Uniejów (**c**), Nowa Wieś (**d**), Śrem (**e**), Poznań (**f**), Skwierzyna (**g**), and Gorzów Wielkopolski (**h**)

To compare the performance of the utilized prediction models, their predictions have been plotted against their measured values in a Taylor diagram (Fig. 7). This comparison was plotted for all the three daily horizons at all stations.

The Taylor diagram provides a visual framework for comparing numerically simulated values with real measurements. It can simultaneously show the error, correlation and standard deviation of the predicted-observed series. As can be seen at first glance, the 1-day ahead prediction points (A [for ANFIS] and B (for ANFIS-ACO) in all the stations) are in the closest positions to observational data points (point O). By increasing the forecasting lead time, the data points move away from point O and in the 3-days ahead prediction points (E [for ANFIS] and F (for ANFIS-ACO) in all stations) they reach their maximum distances. In a specific forecasting horizon, it is clear that the points of ANFIS-ACO (B, D and F) are closer to the O point than those of ANFIS (A, C and E), in all stations; point B is superior to point A, D is superior to C and F is superior to E. The quarter circles relate to standard deviations. The points are close to the O point’s quarter circles, which is more visible in the downstream stations. This indicates that both models accurately estimated the standard deviation of the actual streamflow. Furthermore, in this case, the points of ANFIS-ACO are closer to the quarter circle of the O point than those of ANFIS; which shows that the hybridized ANFIS provides a better standard deviation estimation than its simple form. This difference between ANFIS and ANFIS-ACO is more pronounced in the Bobry, Poznań, and Gorzów Wielkopolski stations. As far as RMSE is concerned, it is obvious that the predictions for the Bobry station are in the best positions, especially point B, which is under the semicircle of RMSE = 2 m^3^/s. In the Sieradz, Uniejów, Nowa Wieś, Śrem and Poznań stations, the predictions vary between the 2 m^3^/s and 4 m^3^/s semicircles of RMSE and in the Skwierzyna and Gorzów Wielkopolski stations, it is out of the RMSE = 4 m^3^/s semicircle. The correlation coefficient (*R*) radiuses indicate that the predictions for the Nowa Wieś, Śrem, Poznań, Skwierzyna and Gorzów Wielkopolski stations have the best correlation with the actual streamflow; thus most of the points in both stations are located under the radius of *R* = 0.99. In the Sieradz and Uniejów stations the data points are mostly located under the radius *R* = 0.95 and finally in Bobry the points are gathered under the radius R = 0.8. Examining these two criteria (RMSE and *R*) presents a contradictory result among the stations which should be further investigated.

### Evaluating the prediction accuracy along the river

**Fig. 8:**
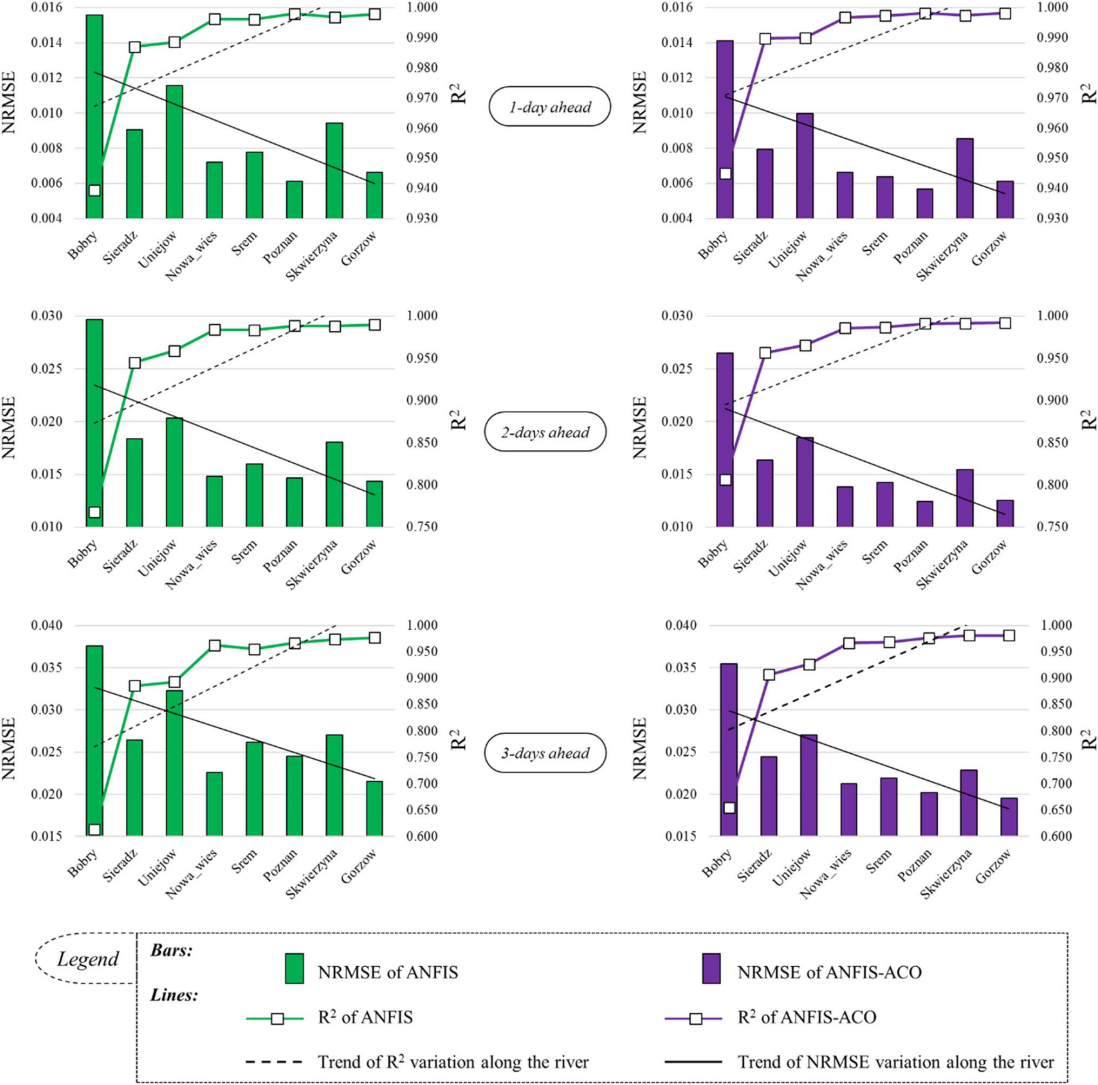
Combo-graphs to evaluate the prediction performance variations along the river, based on the criteria NRMSE and *R*^2^

In this part, the models’ performance along the river was examined. To this end, the NRMSE and *R*^2^ criteria were utilized in all eight hydrometric stations, from the upstream (Bobry) to the downstream (Gorzów Wielkopolski) stations. The results are illustrated as a combination of line and bar charts in Fig. 8.

For all stations, the results of both models were excellent, with NRMSE under 0.1. This evaluation was made for all three prediction lead times. Generally, in all cases (Fig. 8), the smallest *R*^2^ and the largest NRMSE values were recorded for the Bobry station. Moving on to the downstream stations, the *R*^2^ values increased and the NRMSE values decreased, and they reached their best fitted value in the Poznań and Gorzów Wielkopolski stations. From another perspective, by looking at the trend lines, it is clear that there are distinct trends in both NRMSE and *R*^2^ criteria along the river route: a decreasing trend for NRMSE and an increasing trend for *R*^2^ for both models and all prediction lead times. The smallest *R*^2^ (*R*^2^ = 0.613) belongs to the Bobry station for the 3-days lead time, predicted by the non-hybridized ANFIS and the best one belongs to ANFIS-ACO for 1-day ahead prediction for the Poznań and Gorzów Wielkopolski stations (*R*^2^ = 0.998). The NRMSE criterion has its best value in the 1-day ahead prediction by the ANFIS-ACO model (NRMSE = 0.006) in the Poznań and Gorzów Wielkopolski stations, and the worst value was reported for Bobry for the 3-days lead time (NRMSE = 0.038), predicted by the non-hybridized ANFIS method.

### Evaluating the impact of forecasting horizon on prediction accuracy

**Fig. 9:**
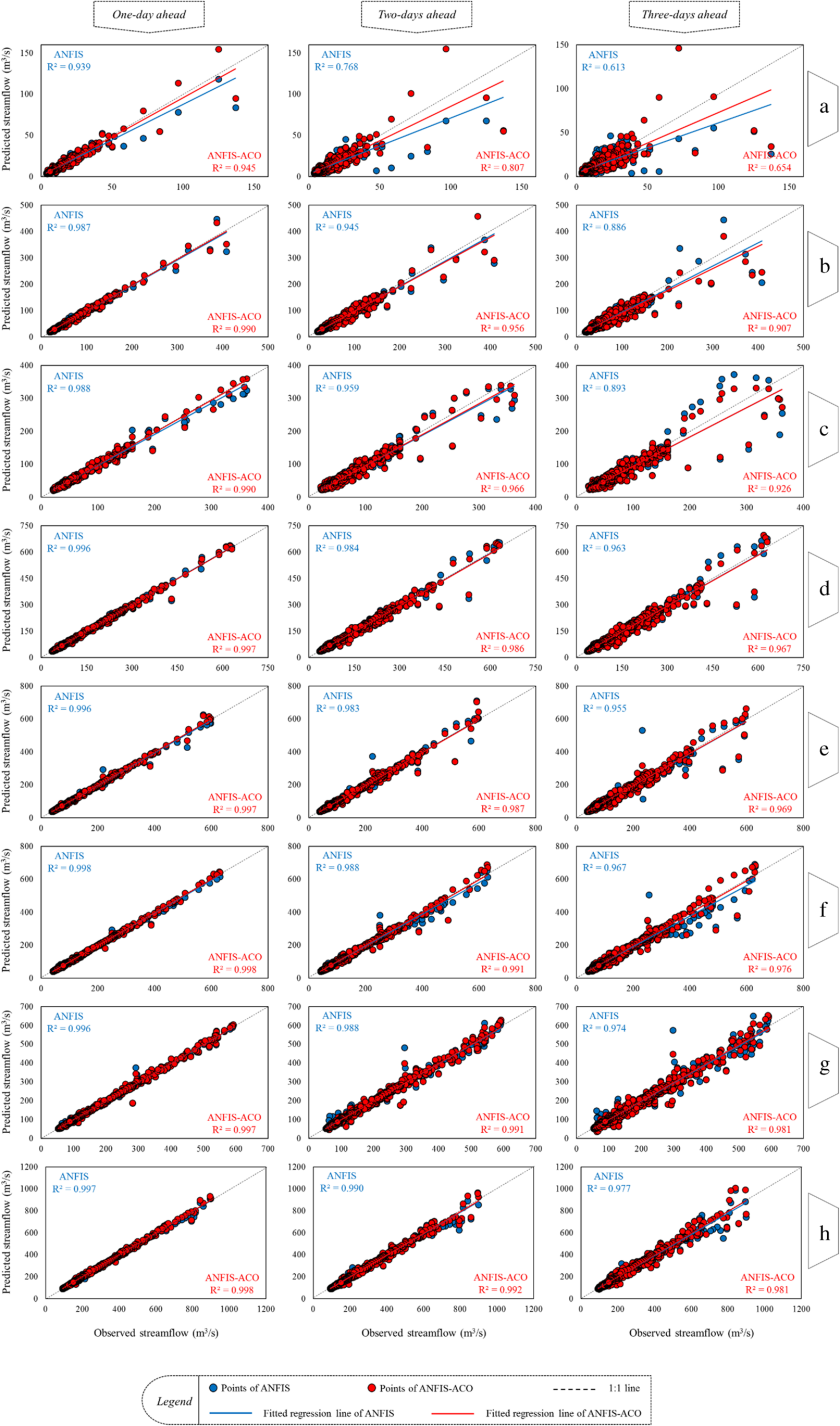
Scatter plots to evaluate the correlation of observed-predicted samples. The small letters on the right side refer to the hydrometric stations, including Bobry (**a**), Sieradz (**b**), Uniejów (**c**), Nowa Wieś (**d**), Śerm (**e**), Poznań (**f**), Skwierzyna (**g**) and Gorzów Wielkopolski (**h**)

To investigate the modes’ prediction accuracy in different daily horizons, scatter plots were produced. For each station, the plots were drawn separately for each lead time. Moreover, in each graph a distinction was made between the performance of ANFIS and that of ANFIS-ACO. The scatter plots are illustrated as Fig. 9.

**Fig. 10:**
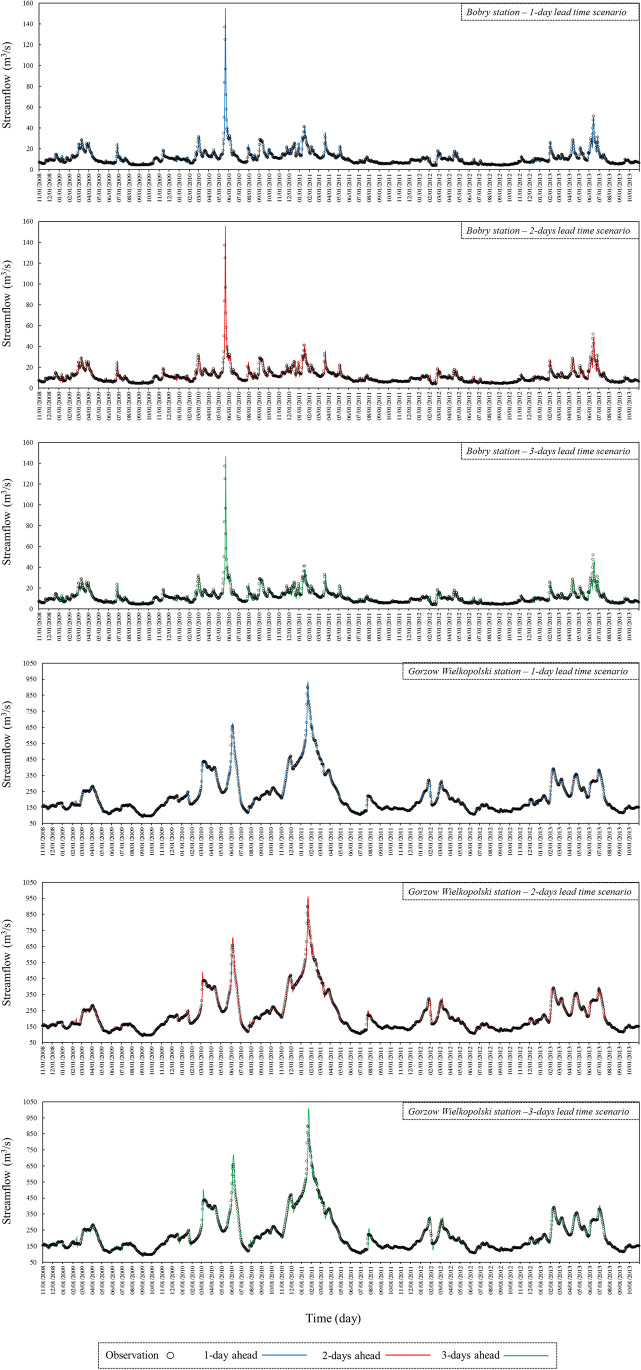
Time series plots to illusterate the overlapping of the observed-predicted series, for the stations Bobry and Gorzów Weilkopolski

It is obvious in the scatter plots (Fig. 9) that the dispersion of the points increases with increasing the forecasting horizon in all hydrometric stations. This indicates that the prediction accuracy decreases when the prediction horizon becomes larger. A decrease of the *R*^2^ value with an increase of the prediction horizon additionally confirms the above finding. The highest *R*^2^ value is equal to 0.998, and belongs to the 1-day lead time of the ANFIS-ACO model in the Poznań and Gorzów Wielkopolski stations. The lowest *R*^2^ value is 0.613, reported for the simple ANFIS model for the 3-days lead time at the Bobry station. In all cases, the linear fitting regression of the ANFIS-ACO model is close to the 1:1 line, which is more pronounced in the upstream stations, especially Bobry, Sieradz and Uniejów. This indicates that the hybridized ANFIS provides a better fitted prediction compared to its simple form. Subsequently, the time series plots were drawn for the three forecasting lead times, for the highest (Bobry) and lowest elevated (Gorzow Wielkopolski) hydrometric stations of the Warta river (Fig. 10). It is worth mentioning that all outputs are related to the ANFIS-ACO model, which had the best prediction performance. The overlapping of the predictions and observations can be better illustrated in this graphical form.

## Discussion

### 1-day ahead streamflow prediction

The prediction of streamflow for 1-day horizon performed by the ANFIS model and its ACO modification generally produced acceptable measures (Table 3). The parameters of ANFIS applied in the training and in the test stage were higher than 0.94 in terms of efficiency (NS) and the highest MAPE was 7.3%. After ACO implementation, prediction accuracy clearly improved both in the training and the test stage. Standard deviation of the predicted streamflow was much closer to the observation series than in the basic version of the algorithm (Fig. 7). A smaller, yet still significant, prediction improvement was observed for *R*^2^ and RMSE. Slightly worse results, following the implementation of the presented methodology, were observed by Azad et al. (2018a) for gauges located on the Zayandehrood River. The mentioned differences are the result of the quite obvious impact of the river regime and hydrometeorological conditions on forecast accuracy. However, it is worth noting that the structure of the differences between the measures of ANFIS and ANFIS-ACO is very similar in both the presented and cited paper, which indicates the wide applicability of this methodology in the area of estimation. Modification of the ANFIS algorithm by ACO also gives promising results with regard to streamflow prediction in a monthly step (Adnan et al. 2022) as well as the qualitative features of river water (Azad et al. 2018b, 2019). Dariane and Azimi (2016) successfully combined two ANFIS methods: subtractive (sub) -ANFIS and fuzzy C-means (FCM) -ANFIS to forecast streamflow in two sub-basins (Lighvan and Ajichai basins) of the Urmia Lake Basin located in Iran. Azad et al. (2018a) used EAs: the GA, ant colony optimization for continuous domain (ACOR) and particle swarm optimization (PSO) to teach ANFIS to predict streamflow. Classic ANFIS was only able to predict streamflow 1-day in advance, while EA improved this ability to 5-days in advance.

In the scatter diagrams for ANFIS and ANFIS-ACO predictions, data pairs in low and middle streamflow sections are located very close to the 45^O^ line which is considered as a very good prediction (Fig. 9). In high streamflow some dispersion appeared, especially in the upstream channel cross-sections between the Bobry and Uniejów water gauges. This seems to be determined by relatively small water resources and catchment area (Table 1) where the reaction of water bodies to hydrometeorological alimentation impulses is much more active and diverse than in the downstream section, characterized by higher water resources, which are more stable and, as a result, easier to predict. The impact of physical and geographical factors of the catchment area and anthropogenic factors on the streamflow of the Warta river, its size and dynamics of changes has been confirmed by the results of research conducted, among others, by Tomaszewski (2001), Wrzesiński (2013) and Wrzesiński and Perz (2016). It is worth noting that in the above- mentioned areas of the scatter plots, the results of the ANFIS model are slightly underestimated whereas the ANFIS-ACO overestimates the prediction with regard to the observed streamflow (Fig. 9).

In the studied river system, a clear tendency towards improved model prediction was observed in the downstream direction (Fig. 8). This is mainly caused by a gradual increase of water resources accompanied by an increased discharge or catchment area (Table 1). The observed process seems to be determined by an increase of alimentation inertia dependent on the differences in river regime between the main river and its tributaries as well as a much more stable reaction of streamflow to replenishment and recession of catchment resources. This observation is also supported by the gradual downstream changes in autocorrelation functions (Fig. 5). Their shape transforms from exponential (Bobry) to linear (Gorzów Wielkopolski), which should be interpreted as an increase in water resources inertia. In three upstream gauging stations (Bobry, Sieradz, Uniejów) a break of the autocorrelation function appeared, more or less close to the lag equal to 20–30 days. This leads to a conclusion that the river system’s “memory” regarding streamflow formation is rather short in the upper part of the Warta Water Region and lasts one month. In the downstream stations this “niche” on the graph fills up due to increasing streamflow inertia.

Some breaks occurred in the trend of model performance improvement in the downstream direction (Fig. 8). The first significant jump of *R*^2^ and NMRSE was observed between the Bobry and Sieradz stations. An increase of the mean streamflow in this river reach was equal to 295% and the rise of catchment area was higher than 350% (Table 1). This indicates a significant progression of water resources, as explained in the previous paragraph, and determines better parameters of model prediction. Another smaller deviation from the general trend was a local increase of NRMSE in the Uniejów station. It is worth noting that in relation to Sieradz, the next station upstream, the average streamflow decreased by approximately 6% (Table 1). This was caused by the dammed Jeziorsko reservoir, located between these two stations (Fig. 1). Water management activity, mainly for energy and agricultural purposes, leads to significant changes in the streamflow regime below the dam and makes the prediction process more complicated. Moreover, partial streamflow depletion due to water abstraction by reservoir users causes some local perturbations in analyses of streamflow alimentation continuity and changes the reference base with respect to average water resources. The fluctuations of the NMRSE in the downstream water gauges seem to be determined by the local water management activity and insignificant fluctuations of river regime, as *R*^2^ is very stable in this reach, ranging between 0.996 and 0.998.

### 2- and 3- day ahead streamflow prediction

The ANFIS model performance goes down along with an increase of the prediction horizon to 2- and 3-days (Tables 4 and 5). The greatest differences in the model accuracy were found in the upstream water gauges (10–20% on average in the whole group of performance measures) whereas in the downstream parts, the forecast quality did not decrease significantly. ACO modifications offer an improvement in the prediction performance proportional to that which was recorded in the 1-day horizon;in the test stage (2-/3-days), the upper station, Bobry, was characterized by NS = 0.81/0.65 and MAPE = 10.76/14.73% whereas the lower water gauge, Gorzów Wielkopolski, reached 0.99/0.98 and 2.29/3.48%, respectively. The relatively significant differences in NS between Bobry and the other downstream hydrological stations (Tables 4 and 5) seem to be determined by relatively small water resources where river reaction to hydrometeorological alimentation impulses is much more active and diverse than in the downstream section. This results in smaller stability of upstream water resources, and consequently, the streamflow is more difficult to predict.

In the scatter diagrams for ANFIS and ANFIS-ACO model predictions, data pairs for 2- and 3-days prediction fit very close to the 45^O^ line in low streamflow only (Fig. 9). Extending the forecast horizon results in the increasing dispersion of data pairs from the high to the middle streamflow zone. On the one hand, this is because high streamflow and flood waves are different in origin (heavy rains, continuous rains, snowmelt, ice jams etc.) and on the other hand, hydrological extremes are relatively rare and more difficult for machine learning. In small catchments (Bobry, Sieradz), extremes are rather short which results in a high dispersion of data pairs (Fig. 8). In middle-sized catchments (from Uniejów to Poznań), a specific kind of loop is observed in high streamflow which reflects the rising and recession phase of significant flood waves. The two lowest water gauges (Skwierzyna, Gorzów Wielkopolski) are characterized by stable and long extremes without high streamflow variability within which the models are able to improve the prediction performance. Changes of *R*^2^ and NRMSE in the ANFIS and ANFIS-ACO models along the river for 2- and 3-days prediction are proportional to the 1-day horizon which suggests that the group of streamflow determiners and their predictions are the same in all cases and the approach as well as the strength of their impact is also similar.

There are two reservoirs along the river that can affect the prediction accuracy of daily streamflow. As is obvious from the autocorrelation graphs (Fig. 5), the autocorrelation is the highest in the first lags and then it decreases. In the upper stations, the first lags are sharper and then they decrease with a greater intensity; while the rate of decrease after the first lag is milder in the downstream stations. Even the first lag has a higher autocorrelation in the more downstream stations, than in the upstream stations. This can be related to the presence of the water reservoirs: the more upstream stations are directly affected by the natural precipitation regime. In the downstream stations, however, the way of releasing water at the outlet of the reservoirs has a time management system that can have a positive effect on the autocorrelation of the daily streamflow data. Accordingly, an increase in the autocorrelation naturally leads to better predictions. The NS criterion is a better tool for understanding this subject, which is shown in Tables 3, 4, and 5. Also, Fig. 8 shows the trend of prediction accuracy better along the river stations: it increases from the upstream to downstream hydrometric stations.

### Practical aspects of using ANFIS-ACO streamflow forecasting models in water resources management

The implementation of new algorithms in the streamflow modeling and forecasting is currently an important direction of research regarding assessment of their potential and the possibility of developing intelligent water resource management systems. On the one hand, the water resources management involves providing community with access to freshwater resources and protection against extreme hydrological phenomena (floods and droughts) to which the Warta river catchment is exposed and on the other hand, ensuring the appropriate ecological condition of waters.

The numerous applications of streamflow models include flood risk assessment and flood /low water forecasting, thus supporting the operational activities of (in Poland) the Institute of Meteorology and Water Management-National Research Institute (IMWM-NRI). The development of official documents concerning flood zones and real-time flood protection is an important field of application of hydrological and hydrodynamic models in Poland. For the purposes of local monitoring and flood protection systems, real-time forecasts are increasingly being used. Operational modeling and forecasting of hydrological processes is one of the tasks of the National Hydrological and Meteorological Service (NHMS) within the IMWM-NRI. In addition, strongly integrated activities with regard to forecasting hydrological processes and phenomena are carried out by hydrological forecast offices (central and regional). Operational hydrological forecasting is largely based on one-dimensional hydrodynamic models which, due to their low complexity, can be used in near-real time. MIKE II is an example of a hydrodynamic model which together with the rainfall-runoff model is used in the Warta river catchment (Ostojski 2013).

The model of daily streamflow forecasts of the Warta river, obtained in the present study for the dataset from the period 1993–2013, can supplement local observation and monitoring systems that primarily support flood prevention activities. Integrated monitoring and local forecasting systems, including modules for short-term streamflow forecasts, could provide local authorities with a reliable tool to support decisions, especially during periods of flood or drought. Owing to the analysis accuracy and a very high forecast accuracy the model can provide an important source of information for crisis management services.

The ANFIS and ANFIS-ACO models are new tools that enable a comprehensive analysis of large and complex databases, and additionally provide an opportunity to supplement expert knowledge with the ability to learn and create new rules. In the case of the Warta river catchment, the following should be taken into account: obtaining better forecasts for the current water gauge profiles, expansion of the river network for which daily forecasts are calculated, extending the range of forecasts to uncontrolled sections, forecasts for periods of floods and hydrological droughts. Besides, the reservoir control module should be included in the model. The Jeziorsko and Poraj reservoirs, located on the Warta river, perform strategic functions. They make it possible to intercept river water during floods and to make up for its deficiencies in the middle and lower section during low flow periods. The Poraj and Jeziorsko reservoirs are multifunctional reservoirs that not only constitute important elements of the flood prevention system, but also perform a maintenance function during periods of low and very low streamflow on the Warta river. In many parts of the Warta Water Region, local disappearance of streamflow and a lowering of the water level in lakes are observed, which pose a serious threat to the protected and valuable natural areas. As a result of cumulative pressures related to water abstraction and area drainage, surface water bodies and protected areas are increasingly vulnerable to the effects of droughts.

Forecasting of daily streamflow on the Warta river using the ANFIS-ACO model is based only on the streamflow in sections of the river system with a 1-, 2-, and 3-days ahead, without taking any precipitation data into account. The investigations have shown that on a daily scale, precipitation has a very weak and insignificant effect on streamflow fluctuations, so it has not been considered as a predictor input. The predictor inputs were selected using the autocorrelation function from among the daily time lags of streamflow for all stations. It can therefore be assumed that this forecast refers to the relationship between the temporary streamflow and water levels that have already occurred in the river system (e.g. water gauges). For Poland (the Vistula River), a similar methodological approach in streamflow forecasting, using neural networks, has been presented by Siuta (2020). Ciszewski and Żelazny (1998) have used a neural model of the rainfall-runoff -water level relationship, Krzanowski and Wałęga (2007) and Licznar (2007) have used artificial neural networks to predict time series of water levels and streamflow, while Siuta (2002) developed a short-term forecast of flood streamflow. Despite the aforementioned examples of applications of the neural network system in streamflow prediction, forecasting modeling of river discharge is rare in Poland. The forecasting model developed for the Warta river does not require much data, but it can provide acceptable streamflow forecasts regardless of the various atmospheric and physical factors that affect it. As the research results show, the models from the proposed ANFIS-ACO group enable better prediction of streamflow, providing a greater accuracy of the results and making the use of the models easy.

## Conclusion

In the present study, the daily streamflow datasets of eight hydrometric stations of the Warta river basin were studied. Two models, i.e., ANFIS in a simple form and ANFIS hybridized with the ACO algorithm, were employed for the prediction of daily streamflow for 1-, 2-, and 3-day lead times. The results showed that the models have acceptable performance in predicting the daily streamflow of the river. According to the NRMSE value, which ranged between 0.016–0.006, 0.030–0.013, and 0.038–0.020 for 1-day, 2-day, and 3-day lead times, respectively, the accuracy of all the predictions was considered to be excellent. The combination of ANFIS with the ACO algorithm enabled to significantly improve streamflow prediction in comparison with a simple ANFIS. This hybridization could averagely increase the prediction accuracies of ANFIS by 12.1%, 12.91%, and 13.66%, for 1-day, 2-days, and 3-day lead times, respectively. In this regard, merging the ACO algorithm with other machine learning models, such as MLP, SVM, and GMDH is recommended to future researchers of this field. Moreover, hybridization of ANFIS with other newly developed optimization algorithms, such as intelligent water drops (IWD), the whale optimization algorithm (WOA), the shuffled frog leaping algorithm (SFLA), etc. is postulated, as it may also have a research value for future studies. It is suggested that this approach could be applied to predict hourly streamflow, and to evaluate the impact of hourly precipitation on streamflow fluctuations. The promising and reliable findings of this approach may have a research value for other similar or different catchment areas, in association with daily streamflow prediction. The current research was designed only based on the time lags of the main variable, i.e., streamflow. Since the surface and meteorological conditions can also affect the changes in river flow, future researchers in this field are recommended to test and evaluate the time lags of other meteorological variables such as temperature, precipitation, pressure, etc. as inputs to the models. It is worth noting that the analysis of model parameters revealed a few relationships with river system characteristics and their spatial variability. On that basis it was possible to assess the influence of some characteristics of river regime, especially regarding the quantity and changeability of water resources and alimentation inertia, on the model estimation accuracy. Autocorrelation analysis and high-low flow model deviation assessment might improve the algorithm’s application to more advanced and specialized goals relating to water management or environmental sciences.

Taking the above findings into account, one should bear in mind the utilitarian nature of the developed models of streamflow forecasts for the Warta river, which can support the operation of systems and operational models used for decision-making purposes by the services appointed for this purpose. The results of the ANFIS and ANFIS-ACO daily streamflow forecast models show a great information potential, which may support the operational hydrology section in hydrological forecast offices in the future in terms of replication and use of the developed data sets in various spatial locations with the possibility of their calibration, verification, and validation at the local level. Fuzzy models of streamflow forecasts have a significant advantage, they need much less information about the hydrological system than conventional probabilistic models, and additionally this information may be fuzzy and imprecise. The ANFIS-ACO model seems to be suitable for modeling complex problems, especially in situations where the relationship between the model factors is unknown. A particular aspect of information concerns the identification of threats and providing warnings, e.g., flood warnings in advance, and information on deepening summer low flows, which is an important indicator of the development of hydrological drought not only in the Warta Water Region, but also in many other catchments and geographical regions. Due to the importance of providing early predictions of streamflow from the flood warning point of view, the current research was designed to predict the streamflow for 1- to 3-days lead times. Considering the optimal accuracy of the model used in these lead times, evaluating its performance for longer lead times (5-days, 7-days, 10-days, etc.) also has research value, which is suggested to future researchers in this field.

## Data Availability

The data are held at the Institute of Meteorology and Water Management-National Research Institute (IMWM-NRI, Warsaw, Poland) and are available at https://danepubliczne.imgw.pl*.*
